# Industrial Systems Biology of *Saccharomyces cerevisiae* Enables Novel Succinic Acid Cell Factory

**DOI:** 10.1371/journal.pone.0054144

**Published:** 2013-01-21

**Authors:** José Manuel Otero, Donatella Cimini, Kiran R. Patil, Simon G. Poulsen, Lisbeth Olsson, Jens Nielsen

**Affiliations:** 1 Department of Chemical and Biological Engineering, Chalmers University of Technology, Gothenburg, Sweden; 2 Center for Microbial Biotechnology, Department of Systems Biology, Technical University of Denmark, Lyngby, Denmark; 3 Department of Experimental Medicine, Second University of Naples, Naples, Italy; Pacific Northwest National Laboratory, United States of America

## Abstract

*Saccharomyces cerevisiae* is the most well characterized eukaryote, the preferred microbial cell factory for the largest industrial biotechnology product (bioethanol), and a robust commerically compatible scaffold to be exploitted for diverse chemical production. Succinic acid is a highly sought after added-value chemical for which there is no native pre-disposition for production and accmulation in *S. cerevisiae*. The genome-scale metabolic network reconstruction of *S. cerevisiae* enabled *in silico* gene deletion predictions using an evolutionary programming method to couple biomass and succinate production. Glycine and serine, both essential amino acids required for biomass formation, are formed from both glycolytic and TCA cycle intermediates. Succinate formation results from the isocitrate lyase catalyzed conversion of isocitrate, and from the α-keto-glutarate dehydrogenase catalyzed conversion of α-keto-glutarate. Succinate is subsequently depleted by the succinate dehydrogenase complex. The metabolic engineering strategy identified included deletion of the primary succinate consuming reaction, Sdh3p, and interruption of glycolysis derived serine by deletion of 3-phosphoglycerate dehydrogenase, Ser3p/Ser33p. Pursuing these targets, a multi-gene deletion strain was constructed, and directed evolution with selection used to identify a succinate producing mutant. Physiological characterization coupled with integrated data analysis of transcriptome data in the metabolically engineered strain were used to identify 2^nd^-round metabolic engineering targets. The resulting strain represents a 30-fold improvement in succinate titer, and a 43-fold improvement in succinate yield on biomass, with only a 2.8-fold decrease in the specific growth rate compared to the reference strain. Intuitive genetic targets for either over-expression or interruption of succinate producing or consuming pathways, respectively, do not lead to increased succinate. Rather, we demonstrate how systems biology tools coupled with directed evolution and selection allows non-intuitive, rapid and substantial re-direction of carbon fluxes in *S. cerevisiae,* and hence show proof of concept that this is a potentially attractive cell factory for over-producing different platform chemicals.

## Introduction

Industrial biotechnology is a promising alternative to traditional petrochemical production of chemicals focused on developing commercially sustainable and environmentally favorable processes [1]. Metabolic engineering, the directed genetic modification of cellular reactions, aims to change the metabolic architecture of microorganisms to efficiently produce target chemicals [2]. Although examples of metabolic engineering successes exist, there has yet to be developed a pipeline where preferred industrial hosts are rapidly engineered to produce a non-native accumulating target metabolite. Recent advances in systems biology enabled *in silico* genome-scale metabolic network reconstructions to guide metabolic engineering strategies [1], [3], [4]. Here we describe a pipeline where a microbial strain was constructed, physiologically characterized, and genomic tools were used to verify the predictions. An essential part of the pipeline is the use of genome-scale metabolic models for initial guiding of the metabolic engineering, which has been shown to be useful also in earlier studies [3], [5], [6]. This approach was repeated and complemented with traditional directed evolution and selection until a proof of concept microbial cell factory was reached. This pipeline resulted in the construction of a non-intuitive *Saccharomyces cerevisiae* cell factory over-producing succinic acid, a building block chemical.

*S. cerevisiae* is the most well characterized eukaryote and is unique in its broad application as an industrial production platform for a large portfolio of products including foods and beverages, bioethanol, vaccines, and therapeutic proteins [1]. Many systems biology tools, including high-throughput genome sequencing, transcriptional profiling, metabolomics, carbon flux estimations, proteomics, *in silico* genome-scale modeling, and bioinformatics driven data integration were first applied to *S. cerevisiae* [4]. Metabolic engineering has benefited from each of these tools; however, relatively few examples exist where cumulative integration has resulted in a generalized pipeline, in particular for the production of a target compound that the organism does not accumulate significantly naturally.

Succinic acid, systematically identified as butanedioic acid (pKa_1_ 4.21, pKa_2_ 5.72), is a value-added chemical building block, with an estimated 15,000 t/year world-wide demand predicted to expand to commodity chemical status with 270,000 t/year [7], [8] representing a potential >2 billion USD annual market. There are several elegant examples of bio-based production of succinate in *Anaerobiospirillium succiniciproducens*, *Actinobacillus succinogenes*, *Succinivibrio dextrinosolvens, Corynebacterium glutanicum, Prevotella ruminocola*, a recently isolated bacterium from bovine rumen, *Mannheimia succiniciproducens*, and a metabolically engineered succinic acid over-producing *E. coli* [7], [8], [9], [10], [11], [12]. All of the hosts described are prokayotic that grow at neutral pH, and consequently secrete the salt, succinate, requiring a cost-intensive acidification and precipitation to reach the desired succinic acid. This concern is not specific to succinic acid production, but rather universal when considering organic acid producing microbial cell factories [13]. *S. cerevisiae* represents a well-established, generally regarded as safe, robust, scalable (1L to 100,000L) industrial production host capable of growth on diverse carbon sources, chemically defined medium, both aerobic and anaerobic, and a wide pH operating range (3.0–6.0). Unlike the hosts described above, succinate is not naturally produced by *S. cerevisiae*; but as there are many factors of importance for the choice of a microbial cell factory it is not uncommon that the chosen cell factory lacks predisposition to produce the target chemical of choice [14]. As industrial biotechnology progresses forward, and the concept of biorefineries are gaining increased importance, platform technologies including microbial cell factories that can plug-and-play into existing infrastructures must be developed [15]. *S. cerevisiae* is uniquely positioned as a platform technology as it is already used widely for bioethanol production, but also because of the extensive library of genetic engineering tools, a very well annotated genome, many omics tools available, and well established complimentary approaches for directed evolution and selection. We therefore addressed the question whether it is possible to metabolically engineer *S. cerevisiae* such that the carbon fluxes are redirected towards succinic acid, and hereby establish proof-of-concept of using this yeast as a general cell factory platform for chemical production. The final strain emerging from this study requires significant further metabolic engineering and process development prior to consideration for commercialization, but the approach and integration of methods demonstrated supports the hypothesis that highly regulated central carbon metabolism in ideal production hosts can be reconfigured to produce target chemicals, relatively quickly and with minimal resources.

## Results

**Figure 1:**
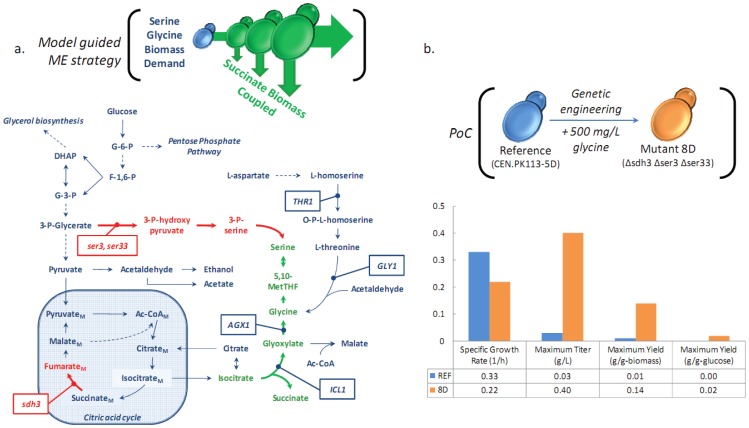
Proof-of-concept: Successful metabolic engineering strategy guided by genome-scale metabolic modeling. Panel a shows the central carbon metabolism of *S. cerevisiae*, and the model-guided metabolic engineering strategy for succinate over-production. Succinate production is directly coupled to biomass formation based on three gene deletions: *sdh3* (cytochrombe b subunit of succinate dehydrogenase complex), and *ser3/ser33* (3-phosphoglycerate dehydrogenase isoenzymes). The remodeling of central carbon flux towards succinate requires minimizing the conversion of succinate to fumarate, and forcing the biomass-required amino acids L-glycine and L-serine to be produced from glyoxylate pools. Production of glyoxylate results from isocitrate conversion by Icl1p, producing equimolar succinate. As the biomass yield increases, the demand for L-glycine and L-serine increase proportionally, driving biomass-coupled succinate production. Legend: native reactions (blue solid line), lumped native reactions (blue dashed line), interrupted reactions (red solid line), up-regulated reactions (green solid line). Panel b demonstrates the proof of concept. The reference strain and genetically engineered mutant strain, 8D, supplemented with 500 mg L**^−^** ^1^ glycine were physiologically characterized in 2L well-controlled stirred-tank fermentations. There was a 13.3X improvement in succinate titer.

In the present study, our objective was to evaluate the use of genome-scale metabolic models for *in silico* identification of gene deletion targets. We therefore used results from a previous study where an evolutionary programming method, termed OptGene, was developed for identification of deletion targets to couple biomass formation (or other biological objective function that is applicable to the organism under question) with the design objective function, such as yield (or other linear/non-linear objective) [16]. These results guided the metabolic engineering strategy described in Figure 1. Glycine, serine, and threonine, all representing essential amino acids required for biomass formation, may be formed from both glycolytic and tricarboxylic acid cycle intermediates. Succinate formation results from the isocitrate lyase, Icl1p, catalyzed conversion of isocitrate to equimolar glyoxylate and succinate, and from the α-keto-glutarate dehydrogenase complex, Kgd1p/Kgd2p/Lpd1p, catalyzed conversion of α-keto-glutarate to equimolar succinate, with a net production of CO_2_, NADH, and ATP. Succinate is subsequently depleted by the succinate dehydrogenase complex, Sdh1p/Sdh2p/Sdh3p/Sdh4p to equimolar fumarate with the net production of protonated ubiquinone. The metabolic engineering strategy identified by OptGene included deletion of the primary succinate consuming reaction encoded by *Sdh3* (cytochrome b subunit of the succinate dehydrogenase complex, essential for function), and interruption of glycolysis derived serine by deletion of 3-phosphoglycerate dehydrogenase, Ser3p/Ser33p (isoenzymes). The remaining pathway for serine synthesis must originate from glycine, and glycine synthesis is largely derived from the alanine:pyruvate aminotransferase, Agx1p, converting glyoxylate and alanine to glycine and pyruvate. With this strategy, glycine and serine biomass requirements are directly coupled to succinate formation via the glyoxylate cycle. Substantial succinate accumulation (defined as >0.1 g L**^−^** ^1^) in the culture broth is not observed in wild-type *S. cerevisiae*, and deletion of *sdh3* has not resulted in appreciable succinate accumulation [17]; a conclusion also found by chemical inhibition of the succinate dehydrogenase complex with titration of malonate (Figure S1), a chemical inhibtor of this complex [18].

**Figure 2:**
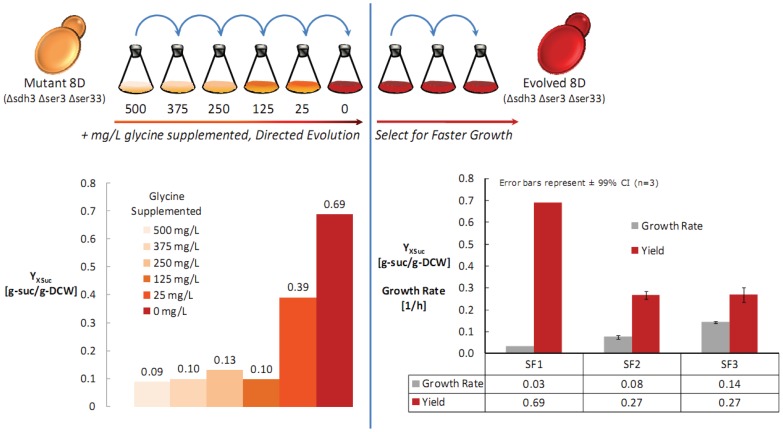
Metabolic engineering enhanced by directed evolutions. Cell populations were transferred across six shake flask cultures until a glycine prototroph was isolated. Subsequently, successive cultures were used to select for faster growth. From the final shake flask (SF3) the strain Evolved 8D was isolated. The succinate yield on biomass is plotted for each shake flask culture, demonstrating a 7.8X increase. The right plot shows the profile of specific growth rate and succinate yield on biomass for the final selection of faster growing cells.

The mutant resulting from the *in silico* strategy, referred to as 8D (*Δsdh3 Δser3 Δser33*), required supplementation with 500 mg L**^−^** ^1^ glycine to be able to grow. When evaluated in well controlled, aerobic, batch stirred tank reactors supplemented with 20 g L**^−^** ^1^ glucose in chemically defined medium, it exhibited a 13-fold improvement in succinate secreted titer (0.03 v 0.40 g L**^−^** ^1^), 14-fold improvement in succinate biomass yield (0.01 v 0.14 g-succinate g-biomass**^−^** ^1^), and a modest 33% reduction in the specific growth rate. Thus, the *in silico* guided metabolic engineering strategy was shown to work, representing a proof-of-concept of the use of model guided metabolic engineering. However, in order to obtain a protothropic strain directed evolution was employed to screen and select for 8D mutants that did not require glycine supplementation. Specifically, six consecutive shake flask cultivations in media supplemented with decreasing concentrations of glycine, from an initial 500 mg L**^−^** ^1^ to 0 mg L**^−^** ^1^ (see Figure 2) were performed. The resulting strain demonstrated a 7.7-fold improvement in succinate yield on biomass (0.09 v 0.69 g-succinate g-biomass**^−^** ^1^), strongly suggesting the direct coupling of glycine formation from glyoxylate and succinate formation. The resulting strain had a relatively low specific growth rate, 0.03 h**^−^** ^1^, and was therefore subsequently cultivated in shake flasks and transferred across six shake flasks (only first three shake flasks shown in Figure 2) to improve the specific growth rate. Finally, a specific growth rate of 0.14 h**^−^** ^1^ was reached, however, resulting in a decreased succinate yield on biomass (0.69 v 0.27 g-succinate g-biomass**^−^** ^1^). The final strain, referred to as 8D Evolved, was shown to exhibit a 60-fold improvement in biomass coupled succinate production (0.01 v 0.30 g-succinate g-biomass**^−^** ^1^), and 20-fold improvement in succinate titer (0.03 v 0.60 g L**^−^** ^1^) relative to the reference strain when grown in aerobic batch cultivations.

To investigate the apparent decoupling of succinate coupled biomass formation, and potentially identify second-round metabolic engineering strategies, the transcriptome was measured in aerobic, glucose-limited, mid-exponential phase grown batch cultivations of 8D Evolved and the reference strain. Continous cultivations, both carbon-limited and nitrogen-limited chemostats were attempted with the 8D Evolved mutant; however, in both cases steady-state was not reached and wash-out occured, even at relatively low dilution rates (*D* = 0.03 h**^−^** ^1^ compared to *µ_max_* = 0.14 h**^−^** ^1^). It was expected that 8D Evolved would not support cultivation in carbon-limited continuous culture due to the down-regulation of the TCA cycle (*Δsdh3*), and consequently, reduced capacity for respiratory metabolism and oxidative phosphorylation. Therefore, batch cultivations were employed acknowledging the significant differences in specific growth rate (0.33 v 0.13 h**^−^** ^1^), and glucose uptake rate (90 v 26 C-mmol g-DCW**^−^** ^1^ h**^−^** ^1^), while maintaining relatively similar biomass yields (0.18 v 0.19 C-mol biomass C-mol glucose**^−^** ^1^).

**Figure 3:**
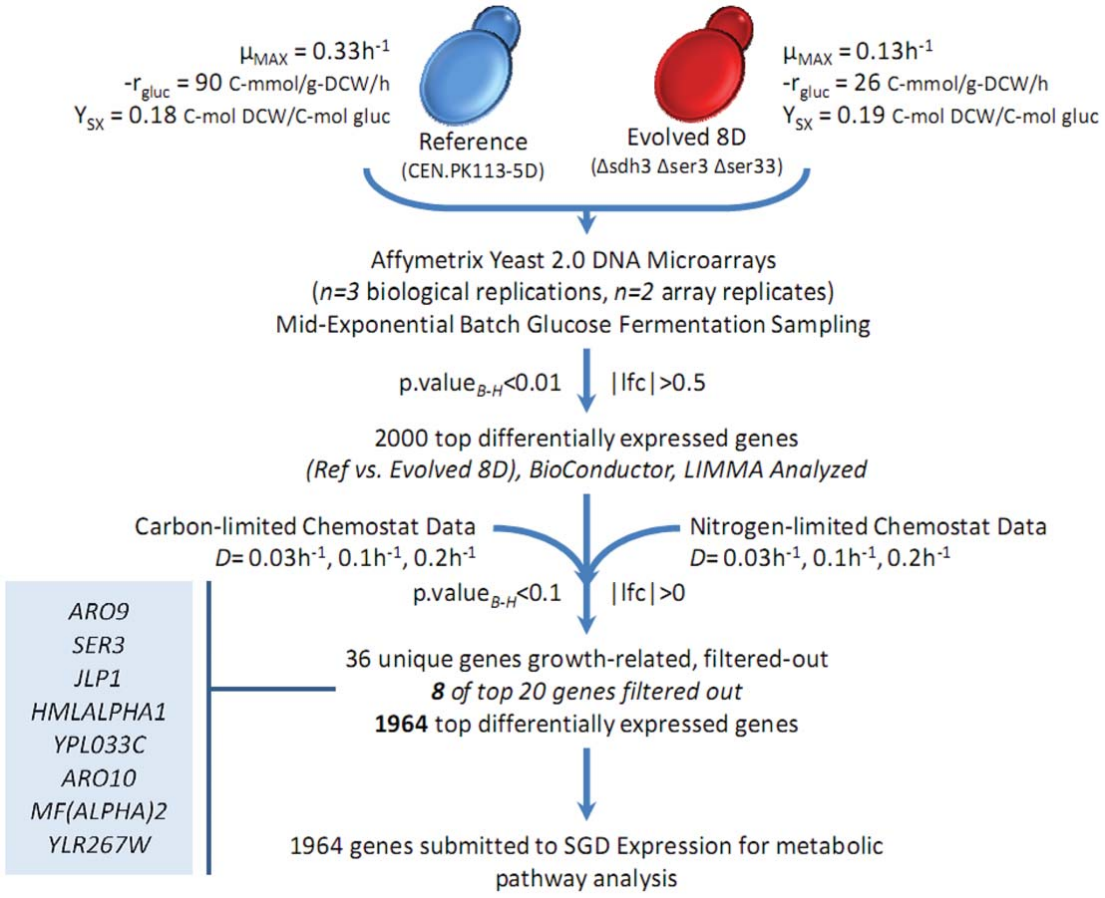
Transcriptome guided metabolic engineering – Analysis. Affymetrix Yeast 2.0 DNA microarrays were used for transcriptome analysis of each strain cultured in well-controlled glucose batch fermentations. The top 2000 differentially expressed genes had an adjusted *p.value<0.01* and *log-fold change (lfc)>0.5*. A carbon-limited and nitrogen-limited chemostat transcriptome data set using the reference strain, surveyed at dilution rates (*D*) of 0.03, 0.1, and 0.2 h**^−^** ^1^ was used to determine which genes are growth-related under each condition. A total of 36 unique growth-related genes were identified from statistical analysis of each data set and with a total of 8 growth-related genes being among the top 20 differentially expressed genes between the reference and evolved 8D strain. After removal of the 36 genes, a total of 1964 genes were carried further for pathway analysis.

Several studies have shown that significant differences in specific growth rate directly impact transcriptome interpretation, with anywhere between 268 and 2400 genes classified as potentially growth-related [19], [20], [21]. Previously generated continuous cultivation transcriptome data for both carbon-limited (glucose, respiratroy growth) and nitrogen-limited (ammonium sulfate, respiro-fermentative growth) conditions at dilution rates of 0.03, 0.1, and 0.2 h**^−^** ^1^ were therefore used to identify statistically differentially expressed growth-related genes [21]. A total of 6 and 7 differentially expressed genes were identified within the carbon-limited and nitrogen-limited data sets as being growth-related (*p-value_B-H_<0.1*, *n = 3* at each dilution rate), respectively, and a total of 66 differentially expressed genes were identified when comparing carbon-limited and nitrogen-limited data sets, paired at each dilution rate (*p-value_B-H_<0.1*, *n = 3* at each dilution rate). Of the total 2406 differentially expressed genes between the 8D Evolved and reference strain (*p-value_B-H_<0.01*, *|log-fold change|>0.5*, *n = 3* biological replicates, *n = 2* DNA microarray duplicates), 36 unique growth-related genes were identified suggesting that few of the genes with a significant change in transcription in 8D Evolved are due to changes in the specific growth rate (see Figure 3). However, a total of 8 of the top 20 *p-value_B-H_* ranked differentially expressed genes identified from pair-wise comparison of 8D Evolved and the reference strain, are growth-related genes (*ARO9, SER3, JLP1, HMALPHA1, ARO10, MFALPHA2,* and two uncharacterized genes, *YPL033c* and *YLR267w*).

The top 2000 (there were no metabolic genes in the remaining 406 genes nor were there any biological process annotations available as determined by gene ontology, and therefore they were not included in further analysis) differentially expressed genes were selected for further analysis, and after removal of the 36 growth-related genes, a list of 1964 genes was submitted for metabolic pathway visualization and characterization to the Expression Viewer [22] available at the Yeast Genome Database [23] (see Figure 3). The log-fold change of the 8D Evolved:Reference expression ratio was mapped onto the metabolic map of *S. cerevisiae* strain S288c, version 12.0, composed of 140 pathways, 925 enzymatic reactions, and a total of 675 compounds (see Figure S2). A total of 315 genes mapped to a specific metabolic pathway on the expression viewer, with a mean log-fold expression ratio value of 0.3±1.3 (*n = 315*, ± SD).

Three biologial insights were immediately apparent (see Figure S2). First, *SDH3*, *SER3*, and *SER33* had negative log-fold expression ratios (log-fold change <-8.0) confirming the gene deletions targeted in the 8D strain and the maintained low expression through the directed evolution. Second, when examining the glycine, serine, and threonine metabolism, *AGX1* was 4.3 log-fold change upregulated in the 8D Evolved strain, confirming significant upregulation of glycine synthesis from glyoxylate pools, as predicted by the original metabolic engineering strategy. However, there was no upregulation of *SHM2, SHM1*, the genes encoding pathways for L-serine formation from L-glycine pools. Most surprisingly *GLY1*, encoding threonine aldolase, was significantly up-regulated (log-fold change 1.6). In the genome-scale metabolic network reconstructions of *S. cerevisiae* iFF708, iND750, and iIN800, upon which the 8D metabolic engineering strategy is based, Gly1p encodes the irreversible conversion of glycine and acetaldehyde to threonine [24], [25], [26], leading to the prediction that threonine biosynthesis from glycolytic intermediates could be down-regulated, and provided for from glycine pools. This consequently leads to a greater biomass-coupled drive for glyoxylate synthesis from isocitrate, yielding equimolar succinate. Levaraging this over-all strategy, another *S. cerevisiae* mutant was constructed, referred to as 20G (*Δsdh3, Δser3, Δthr1*), where Thr1p, encoding homoserine kinase that is required for threonine biosynthesis, was deleted. However, this strain required threonine supplementation and after several extensive attempts at adaptive evolution, the threonine auxotrophy persisted, suggesting the irreversibility of *Gly1* was incorrect and the aldolase strongly favors glycine formation (Figure S3). The significant up-regulation of *Gly1* therefore provides a strong hypothesis for why 8D Evolved had an attenuation of succinate production, even under increasing specific growth rate, suggesting a decoupling of biomass coupled succinate production. It should be noted that in the most recent update of the genome-scale metabolic reconstruction of *S. cerevisiae*, iMM904, the directionality of *Gly1* was corrected to now indicate threonine irreversible conversion to glycine and acetaldehyde [27].

**Figure 4:**
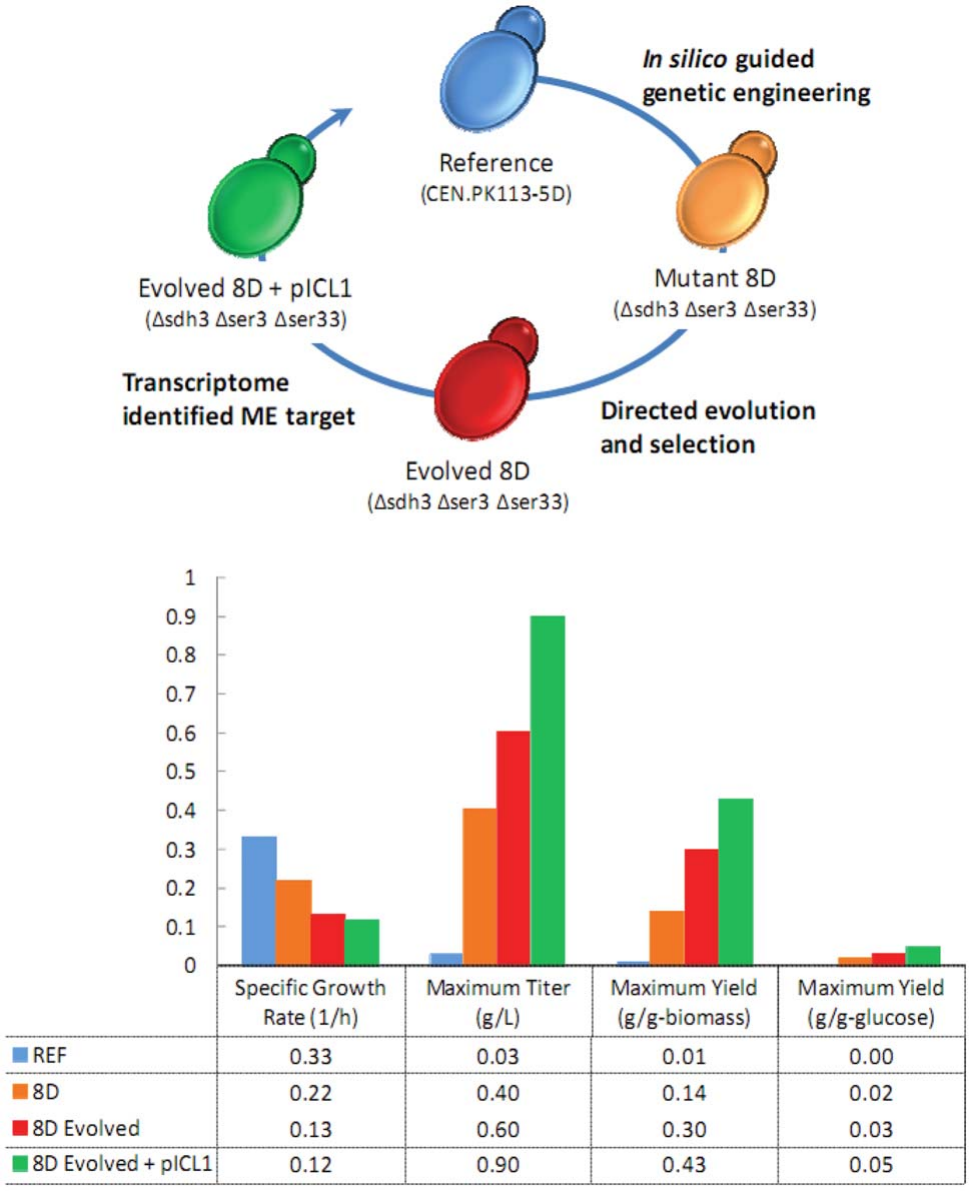
Summary of succinate microbial cell factory construction. The specific growth rate (1/h), maximum succinate titer (g/L), maximum succinate yield on biomass (g/g-biomass), and maximum yield on glucose (g/g-glucose) are reported for the reference strain, 8D, 8D evolved, and 8D evolved with pICL1. A 43-fold improvement in succinate yield on biomass was observed across the full cycle of metabolic engienering that included *in silico* guided approaches, directed evolution, and transcriptome based identification of a 2^nd^ round of metabolic engineering targets.

The transcriptome not only provides for a global, rapid, and quantitative assessment of the predicted *in silico* metabolic engineering strategy and insight into the genetic engineering modifications that result from directed evolution and selection, but also provides a source for identification of second round metabolic engineering targets not previously predicted. Several targets were identified, but of particlar interest was *ICL1*, encoding isocitrate lyase, converting isocitrate to glyoxylate and succinate in equimolar concentrations. All tricarboxylic acid cycle genes are up-regulated, with the exception of *SDH3* (target gene deletion), and *ICL1*, providing a clear metaboic engineering target for up-regulation in the 8D Evolved strain. Therefore, native *ICL1* was PCR amplified and cloned into the 2 µm ori plasmid pRS426CT containing the strong constitutive *TEF1* promoter and *CYC1* terminator [28], and then transformed into the reference, 8D, and 8D Evolved strain (strains transformed with the constructed plasmid pRS426T-ICL1-C are referred to as “with pICL1”). All strains were evaluated in aerobic, glucose-supplemented batch fermentations, and only 8D Evolved with pICL1 exhibited a change in succinate production (see Figure 4). Specifically, the succinate titer, biomass yield, and glucose yield were 0.90 g L**^−^** ^1^, 0.43 g-succinate g-biomass**^−^** ^1^, and 0.05 g-succinate g-glucose**^−^** ^1^, respectively, representing a 1.5-fold, 1.4-fold, 1.7-fold improvement over 8D, respectively (see Figure 4).

## Discussion

A *S. cerevisiae* strain capable of succinate production, requiring redirection of carbon flux from typically produced C_2_ (ethanol, acetate) and C_3_ (glycerol, pyruvate) over-flow metabolites to the target C_4_ succinic acid was achieved through metabolic engineering, requiring integration of systems biology methods and directed evolution. Clearly, the resulting strain (8D Evolved with pICL1), while being a successful demonstration of a multi-round metabolic engineering strategy, still requires significant process development and further enhancement to compete commercially with existing bacterial platforms.

The resulting strain, 8D Evolved with pICL1, represents a 30-fold improvement in succinate titer, and a 43-fold improvement in succinate yield on biomass, with only a 2.8-fold decrease in the specific growth rate compared to the reference strain. Despite success of using simple stoichiometric-based calculations for driving metabolic engineering, it is interesting to note that regulatory mechanisms not captured in these models are likely playing a significant role in the succinate production observed. The biomass requirements for glycine and serine are 0.290 and 0.185 mmol g-DCW**^−^** ^1^ [24]. Assuming that all glycine, and all glycine and serine combined demands are supplied from the glyoxylate pool, then the theoretical production of succinate would amount to 0.034 and 0.056 g-succinate g-DCW**^−^** ^1^, respectively. The 8D and 8D Evolved strains are producing 0.30 and 0.43 g-succinate g-biomass**^−^** ^1^, respectively, suggesting a nearly 8-fold higher succinate production than required to meet biomass amino acid demands. A potentially 3^rd^ metabolic engineering target would be deletion of *GLY1* to further minimize alternative biosynthetic routes of glycine production, thereby isolating all glycine production to be dependent on glyoxylate formation, and consequently succinate formation. Yet, it’s clear that any increase in succinate formation would not be due to biomass requirements, but rather regulatory (e.g., non-stoichiometric driven) mechanisms. Therefore, while the strategy presented and demonstrated here is likely to be a major component of an over-all succinate production cell factory, complimentary strategies focussing on the other major succinate production pathway, TCA cycle, will be required. Examples of malic acid production, that included engineering of pyruvate carboxylation (overexpression of *PYC2*), oxaloacetate reduction (overexpression of *MDH3*), and malate export (functional expression of the non-native Sp*MAE1*), resulted in a *S. cerevisiae* strain capable of producing 59 g-malate L**^−^** ^1^ and 0.42 mol malate mol-glucose**^−^** ^1^ [29]. A similar approach, requiring yet further engineering and understanding of the reductive TCA cycle to convert malate to succinate is likely required, but a major hurdle with this strategy is the conversion of fumarate to succinate by fumarate reductase which is thermodynamically favoured in the direction of fumarate.

The transcriptome analysis performed, specifically consideration of continous culture data sets at different dilution rates to filter growth-related genes was an integral part of identifying the 2^nd^ round of metabolic engineering targets. Although a relatively small number of growth related genes were identified, they were of high-value. For example, *ARO9* and *ARO10*, encoding key enzymatic conversion steps in aromatic amino acid metabolism may have incorrectly pointed towards tryptophan, tyrosine, and phenylalanine catabolism or phosphenolpyruvate decarboxylase activity as metabolic areas of interest for understanding physiological differences between 8D Evolved and the reference strains. This approach may be extended to future efforts and other organisms, where continuous cultivation of engineered strains may not be possible, as in this case, or applied industrially where the dominant and preferred processing mode is batch.

Furthermore, this work clearly demonstrated that obvious genetic targets did not result in increased succinate formation. Specifically, deletion of the primary succinate consuming pathway (*Δsdh3*) [17], or constitutive over-expression of one of two of the primary succinate formation pathways (*ICL1*) did not result in any increased succinate production (See Figure S1). It is further interesting to note that the 8D with pICL1 strain also did not result in any increased succinate production, but rather only in the 8D Evolved with pICL1 strain. The ability to measure transcriptome on a strain that underwent targetted genetic engineering and directed evolution was critical to identifying pICL1 as a 2^nd^ metabolic engineering target, which would have been discarded if selected based on intuition.

The approach employed represents an integration of diverse methods for rapid metabolic engineering proof-of-concept. The strain selection process thus need not be limited to considering organisms showing a predisposition to the production of the metabolite of interest, but rather, should include hosts most suitable for large-scale, robust, and biorefinery processing. With such hosts, carbon and redox flux redistribution requiring multi-gene approaches can be predicted, tested, and supplemented with directed evolution, screening, and selection. These strains are then transcriptionally characterized and optimized until commercially viable titers, productivities, and yields are reached. It is only through whole-process optimization and elimination of severe constraints such as forced use of non-industrially favorable strains, that the promise of a bio-based economy may be fully realized.

## Materials and Methods

### Strain Construction

The reference strain *Saccharomyces cerevisiae* CEN.PK113-5D (*Mat a MAL2-8C SUC2 URA3-52*) [30] was used for the construction of the *Δsdh3 Δser3 Δser33* knockout strain, referred to as the 8D mutant, and for the construction of the *Δsdh3 Δser3 Δthr1* knockout strain, referred to as the 20G mutant, through the cloning-free PCR-based allele replacement method previously described [31]. The upstream *SDH3* fragment was amplified by PCR from genomic DNA using the primers SDH3_Up_Fw (sequence 5′-CGAAATATGGTAAGAGAAAATG-3′) and SDH3_Up_Rv (sequence 5′- CAGGGATGCGGCCGCTGACGACATCG TTTATTATTCTTAGAGC-3′). Similarly, the downstream *SDH3* fragment was amplified using the primers SDH3 _Dw_Fw (sequence 5′- CCGCTGCTAGGCGCGCCGTGCTTTATGATTCTTTAAGGCGACGC-3′) and SDH3_Dw_Rv (sequence 5′- GTAATCTGTTATCGATAATCTGCC -3′). The upstream *THR1* fragment was amplified by PCR from genomic DNA using the primers THR1_Up_Fw (sequence 5′-GCAGTTC TTGCTCAGTAATCTTAG-3′) and THR1_Up_Rv (sequence 5′-GCAGGGATGCGGCCGCTGACCCATA TCTTTCGAGATGATGACTC-3′). Similarly, the downstream *THR1* fragment was amplified using the primers THR1 _Dw_Fw (sequence 5′-CCGCTGCTAGGCGCGCCGTGCATACTGTAATTGACCGTTAACGG-3′) and THR1_Dw_Rv (sequence 5′- CCAATCATGGATGAACCAGTAATG-3′). The upstream *SER3* fragment was amplified by PCR from genomic DNA using the primers SER3_Up_Fw (sequence 5′- CTCACAATCGAGTAA TGCCTTTG-3′) and SER3_Up_Rv (sequence 5′- GCAGGGATGCGGCCGCTGACCATTGCTGTCGA TTTTTCTGTGG-3′). Similarly, the downstream *SER3* fragment was amplified using the primers SER3 _Dw_Fw (sequence 5′- CCGCTGCTAGGCGCGCCGTGGGATAGAAGAATGCTTGAGGC-3′) and SER3_Dw_Rv (sequence 5′- CGAATTTGATTGTACCTGGTGC-3′). The upstream *SER33* fragment was amplified by PCR from genomic DNA using the primers SER33_Up_Fw (sequence 5′- GTACTCTTTATGGGAGTCTTTAGC -3′) and SER33_Up_Rv (sequence 5′- GCAGGGATGCGGCCGCTGACGCAGCTGAATAAGACATGTTAGG- 3′). Similarly, the downstream *SER33* fragment was amplified using the primers SER33 _Dw_Fw (sequence 5′- GCAGGGATGCGGCCGCTGACGCAGCTGAATAAGACATGTTAGG- 3′) and SER33_Dw_Rv (sequence 5′-CTATT CTGGGTGGTCTTTTACTGG- 3′). The lithium acetate tranformation method was used [32]. As described previously, *URA3* from *Kluyvermyces lactis* was used as the selection marker in the transformation process [31]. With this approach tranformants are easily selected on uracil depleted media supplemented with 5-fluoroorotic acid. The knockout was confirmed by restriction analysis followed by sequencing (MWG Biotech AG, Ebersberg, Germany).

The plasmid pRS426T-ICL1-C was constructed and transformed into 8D Evolved, described earlier and used for constitutive *S. cerevisiae ICL1* overexpression. The parent plasmid, pRS426CT (6347 bp), was previously constructed in our laboratory by inserting the strong constitutive *TEF1* promoter (gene encoding *S. cerevisiae* translation-elongation factor 1α) and the *CYC1* transcription terminator into pRS426 [28]. This original backbone plasmid is a 5726 bp yeast episomal plasmid (YEp)-type shuttle vector with a high copy number of about 20 per cell [33]. The plasmid contains the 2 µm ori and pUC ori for independent episomal replication in *S. cerevisiae* and *E. coli*, respectively, and *URA3* and *ampR* (*bla*, beta-lactamase) genes. The final plasmid size was 8074 bp, with 2484 containing the *TEF1* promoter, the *ICL1* insert, and the *CYC1* transcription terminator sequence, verified by sequencing (MWG Biotech AG, Ebersberg, Germany).

A total of eight primers were required for amplification of the native *ICL1* gene from the reference strain, sequencing of the constructed plasmid pRS426-ICL1-C, and PCR to verify plasmid presence in the transformed reference and 8D Evolved strains (referred to as 8D Evolved with pICL1). The PCR amplification of *ICL1* was carried out using the Phusion™ High-Fidelity DNA Polymerase (Finnzymes Oy, Espoo, Finland) according to the manufacturer’s protocol. The native *ICL1* was amplified from genomic DNA using the up- and downstream primers ICL1_Sp1 (sequence 5′-GCCTGCCA|CTAGTCAACGAAAAATGCCTATCCCCG-3′), and ICL1_Asp1 (sequence 5′-GCCTCGACCCGGGCTAGAGAAAGGCATTCTTGCACGG-3′ ), respectively. The amplicon length was 1915 bp. The fragment was cut with restriction endonucleases (REN) *Spe*I, the restriction site of which was *de novo* introduced on primer ICL1_Sp1, and *Ngo*MIV, and then ligated with pRS426CT cut with *Spe*I and *Xma*I. By using the non-compatible RENs in either end of the insertion, the direction of the insert is secured and furthermore the sole parent plasmid *Xma* site is lost. This allowed for an *in vitro* pre-selection for the correct pRS426-ICL1-C construct prior to transformation.

The four sequencing primers for construct verification included M13_rev_-29 (sequence 5′-CAGGAAACAGCTATGACC-3′), ICL1_In_1f (sequence 5′-CTGGTTGGCAGTGTTCATCA-3′), ICL1_In_2f (sequence 5′-CATCCCACAGAGAAGCCAAG-3′), and M13_uni_-21 (sequence 5′-TGTAAAACGACGGCCAGT-3′). The two primers used for plasmid verification via PCR (Taq DNA Polymerase of *Thermus aquaticus* from Sigma, St. Louis, MO, were ICL1_part_Sense (sequence 5′-TCCTGTTCAGATTTCTCAAATGGC-3′) and ICL1_CYC_Antisense (sequence 5′-AAATTAAAGCCTTCGAGCGTCCC-3′) and these were used for analytical PCRs according to the instruction manual’s recommendations). Plasmid transformation of electrocompetent *E. coli* DH5α were completed as described previously, as was plasmid transformation of the *S. cerevisiae* reference strain and 8D Evolved using the lithium acetate method [28], [31], [32].

### Medium Formulation

A chemically defined minimal medium of composition 5.0 g L**^−^** ^1^ (NH_4_)_2_SO_4_, 3.0 g L**^−^** ^1^ KH_2_PO_4_, 0.5 g L**^−^** ^1^ MgSO_4_•7H_2_O, 1.0 mL L**^−^** ^1^ trace metal solution, 300 mg L**^−^** ^1^ uracil, 0.05 g L**^−^** ^1^ antifoam 204 (Sigma-Aldrich A-8311), and 1.0 mL L**^−^** ^1^ vitamin solution was used for all shake flask and 2L well-controlled fermentations [34]. The trace elment solution included 15 g L**^−^** ^1^ EDTA, 0.45 g L**^−^** ^1^ CaCl_2_•2H_2_O, 0.45 g L**^−^** ^1^ ZnSO_4_ •7H_2_O, 0.3 g L**^−^** ^1^ FeSO_4_•7H_2_O, 100 mg L**^−^** ^1^ H_3_BO_4_, 1 g L**^−^** ^1^ MnCl_2_•2H_2_O, 0.3 g L**^−^** ^1^ CoCl_2_•6H_2_O, 0.3 g L**^−^** ^1^ CuSO_4_•5H_2_O, 0.4 g L**^−^** ^1^ NaMoO_4_•2H_2_O. The pH of the trace metal solution was adjusted to 4.00 with 2 M NaOH and heat sterilized. The vitamin solution included 50 mg L**^−^** ^1^ d-biotin, 200 mg L**^−^** ^1^ *para-*amino benzoic acid, 1 g L**^−^** ^1^ nicotinic acid, 1 g L**^−^** ^1^ Ca•pantothenate, 1 g L**^−^** ^1^ pyridoxine HCl, 1 g L**^−^** ^1^ thiamine HCl, and 25 mg L**^−^** ^1^ m•inositol. The pH of the vitamin solution was adjusted to 6.5 with 2 M NaOH, sterile-filtered and the solution was stored at 4°C. The final formulated medium, excluding glucose and vitamin solution supplementation, is adjusted to pH 5.0 with 2 M NaOH and heat sterilized. For carbon-limited cultivations the sterilized medium is supplemented with 20 g L**^−^** ^1^ glucose, heat sterilized separately, and 1.0 mL L**^−^** ^1^ vitamin solution is added by sterile filtration (0.20 µm pore size Ministart®-Plus Sartorius AG, Goettingen, Germany). For cultures where glycine or threonine auxotrophic strains were cultivated the final culture medium was supplemented with glycine 500 mg L**^−^** ^1^ or 100 mg L**^−^** ^1^ threonine added by sterile filtration.

### Shake Flask Cultivations and Stirred Tank Fermentations

Shake flask cultivations were completed in 500 mL Erlenmeyer flasks with two diametrically opposed baffles and two side-necks with septums for sampling by syringe. Flasks were heat sterilized with 100 mL of medium, inoculated with a single colony, and incubated at 30°C with orbital shaking at 150 RPM. Stirred tank fermentations were completed in well-controlled, aerobic, 2.2L Braun Biotech Biostat B fermentation systems with a working volume of 2L (Sartorius AG, Goettingen, Germany). The temperature was controlled at 30°C. The fermenters were outfitted with two disk-turbine impellers rotating at 600 RPM. Dissolved oxygen was monitored with an autoclavable polarographic oxygen electrode (Mettler-Toledo, Columbus, OH). During aerobic cultivation the air sparging flow rate was 2 vvm. The pH was kept constant at 5.0 by automatic addition of 2 M KOH. Off-gas passed through a condenser to minimize the evaporation from the fermenter. The fermenters were inoculated from shake flask precultures to an initial OD_600_ 0.01.

### Fermentation Analysis

#### Off-gas Analysis

The effluent fermentation gas was measured every 30 seconds for determination of O_2_(g) and CO_2_(g) concentrations by the off-gas analyzer Brüel and Kjær 1308 (Brüel & Kjær, Nærum, Denmark).

#### Biomass Determination

The optical density (OD) was determined at 600 nm using a Shimadzu UV mini 1240 spectrophotometer (Shidmazu Europe GmbH, Duisberg, Germany). Duplicate samples were diluted with deionized water to obtain OD_600_ measurements in the linear range of 0–0.4 OD_600_ Samples were always maintained at 4°C post-sampling until OD_600_ and dry cell weight (DCW) measurements were performed. DCW measurements were determined through the exponential phase, until stationary phase was confirmed according to OD_600_ and off-gas analysis. Nitrocellulose filters (0.45 µm Sartorius AG, Goettingen, Germany) were used. The filters were pre-dried in a microwave oven at 150W for 10 min., and cooled in a dessicator for 10 min. 5.0 mL of fermentation broth were filtered, followed by 10 mL DI water. Filters were then dried in a microwave oven for 20 min. at 150W, cooled for 15 min. in a desiccator, and the mass was determined.

#### Metabolite Concentration Determination

All fermentation samples were immediately filtered using a 0.45 µm syringe-filter (Sartorius AG, Goettingen, Germany) and stored at −20°C until further analysis. Glucose, ethanol, glycerol, acetate, succinate, pyruvate, fumarate, citrate, oxalate, and malate were determined by HPLC analysis using an Aminex HPX-87H ion-exclusion column (Bio-Rad Laboratories, Hercules, CA). The column was maintained at 65°C and elution performed using 5 mM H_2_SO_4_ as the mobile phase at a flow rate of 0.6 mL min. **^−^** ^1^. Glucose, ethanol, glycerol, acetate, succinate, citrate, fumarate, malate, oxalate were detected on a Waters 410 differential refractometer detecter (Shodex, Kawasaki, Japan), and acetate and pyruvate were detected on a Waters 468 absorbance detector set at 210 nm.

### Transcriptomics

#### RNA Sampling and Isolation

Samples for RNA isolation from the late-exponential phase of glucose-limited batch cultivations were taken by rapidly sampling 25 mL of culture into a 50 mL sterile Falcon tube with 40 mL of crushed ice in order to decrease the sample temperature to below 2°C in less than 10 seconds. Cells were immediately centrifuged (4000 RPM at 0°C for 2.5 min.), the supernatant discarded, and the pellet frozen in liquid nitrogen and it was stored at −80°C until total RNA extraction. Total RNA was extracted using the FastRNA Pro RED kit (QBiogene, Carlsbad, USA) according to manufacturer’s instructions after partially thawing the samples on ice. RNA sample integrity and quality was determined prior to hybridization with an Agilent 2100 Bioanalyzer and RNA 6000 Nano LabChip kit according to the manufacturer’s instruction (Agilent, Santa Clara, CA).

#### Probe Preparation and Hybridization to DNA Microarrays

Messenger RNA (mRNA) extraction, cDNA synthesis, labeling, and array hybridization to Affymetrix Yeast Genome Y2.0 arrays were performed according to the manufacturer’s recommendations (Affymetrix GeneChip® Expression Analysis Technical Manual, 2005–2006 Rev. 2.0). Washing and staining of arrays were performed using the GeneChip Fluidics Station 450 and scanning with the Affymetrix GeneArray Scanner (Affymetrix, Santa Clara, CA).

#### Microarray Gene Transcription Analysis

Affymetrix Microarray Suite v5.0 was used to generate CEL files of the scanned DNA microarrays. These CEL files were then processed using the statistical language and environment R v5.3 (R Development Core Team, 2007, www.r-project.org), supplemented with Bioconductor v2.3 (Biconductor Development Core Team, 2008, www.bioconductor.org) packages Biobase, affy, gcrma, and limma [35], [36]. The probe intensities were normalized for background using the robust multiarray average (RMA) method only using perfect match (PM) probes after the raw image file of the DNA microarray was visually inspected for acceptable quality. Normalization was performed using the qspline method and gene expression values were calculated from PM probes with the median polish summary. Statistical analysis was applied to determine differentially expressed genes using the limma statistical package. Moderated *t-*tests between the sets of experiments were used for pair-wise comparisons. Emperical Bayesian statistics were used to moderate the standard errors within each gene and Benjamini-Hochberg’s method was used to adjust for multi-testing. A cut-off value of adjusted *p*<0.05 was used for statistical significance. Furthermore, principal component analysis (PCA) was performed in order to elucidate the relative importance of substrate limitation (carbon vs. nitrogen) and growth rate (0.03 h**^−^** ^1^, 0.1 h**^−^** ^1^, 0.2 h**^−^** ^1^), previously described [21], when compared with the gene expression of the reference and 8D Evolved strain. To select genes whose expression levels were related to these factors, the moderated *t-*statistics were followed up with *F-*distributions to yield a statistic referred to as *F_g_,* which is simply the usual *F-*statistic from linear model theory but with the posterior variance substituted for the sample variance in the denominator, as described else where [35]. The cut-off value of adjusted *p*<0.1 was used for statistical significance.

All microarray data is MIAME compliant and the raw data has been deposited in ArrayExpress (http://www.ebi.ac.uk/microarray-as/ae/).

## Supporting Information

Figure S1 **Inhibition of the succinate dehydrogenase complex with malonate supplementation in shake flask cultures was evaluated.** The reference and *Δsdh3* strain, previously described [17], were cultured in minimal media supplemented with 10 g L^−1^ glucose and no succinate accumulation was detected (Panel a). The reference strain was cultured with 0.1, 1.0, 5.0, 10.0, and 50.0 mM malonate supplementation. Under no supplementation conditions succinate accumulation was observed (Panel b). In order to confirm that the concentration of malonate in the culture was effectively inhibiting succinate dehydrogenase activity, residual ethanol in the culture broth was monitored. Succinate dehydrogenase activity, as previously described, catalyzes the conversion of succinate to fumarate with net production of protonated ubquinone. Ethanol is a carbon source readily catabolized by *S. cerevisiae* using respiro-fermentative pathways and requiring succinate dehydrogenase activity. Panel c shows the residual glucose concentration in the culture broth at 0, 17, 22, and 37h post-inoculation for no supplementation of malonate (reference) and then 0.1, 1.0, 5.0, 10.0, and 50.0 mM malonate supplementation. These growth profiles were generated using the reference strain (CEN.PK113-7D). As expected, full catabolism of glucose was observed at all malonate concentrations with the exception of 50.0 mM, thereby considered an upper limit. Similarly, in panel d, is the ethanol concentration in the culture broth for the same malonate concentrations and sample times. At 37 h, as expected, the reference strain had consumed nearly all ethanol produced during the glucose consumption phase. Malonate concentrations of 1.0, 5.0, and 10.0 mM malonate resulted in significant ethanol respiration inhibition compared to no supplementation and 0.1 mM malonate, confirming that respiro-fermentative catabolism was inhibited. Under no circumstances was succinate accumulation observed. Furthermore, the *Δsdh3* strain was supplemented with 50.0 mM malonate to ensure no unexpected interaction between the genetic modification and malonate supplementation (panel e).
(PNG)

Figure S2 **A total of 1964 genes were submitted to the Saccharomyces Genome Database tool, Pathway Expression Viewer.** The resulting Pathway Expression map shows the relative log-fold change of all *S. cerevisiae* metabolic reactions (Evolved 8D vs. Reference). Three key results are high-lighted from the transcriptome. First, isocitrate lyase (*ICL1*) was amongst the few genes not up-regulated in the Evolved 8D strain, thereby becoming a 2^nd^ round metabolic engineering target. Second, alanine:glyoxylate aminotransferase (*AGX1*) was 4.3 log-fold higher in the Evolved 8D strain, confirming the predicted model-guided strategy of up-regulated glycine formation from glyoxylate pools. Third, threonine aldolase (*GLY1*) was 1.6 log-fold higher in the Evolved 8D strain. The genome-scale model reconstruction used for predictions annotated Gly1p as catalyzing the reversible conversion of threonine to glycine. This reaction has since been shown to be irreversible, converting threonine to glycine, consuming equimolar acetaldehyde. The transcriptome data suggests that the Evolved 8D strain demonstrated de-coupling of succinate and biomass production because alternative reactions (e.g., Gly1p) were supplying glycine pools.
(PNG)

Figure S3 **Panel a briefly describes the mutant construction of 20G,** ***Δsdh3 Δser3 Δthr1*** **, from the reference strain and initially supplemented with 100 mg L^−1^ threonine and 500 mg L^−1^ glycine to satisfy the resulting auxotrophies.** All growth challenges were evaluated in shake flasks supplemented with minimal medium, 300 mg L**^−^** ^1^ uracil, 10 g L**^−^** ^1^ glucose, and either threonine and/or glycine added, as indicated. The mutant 20G was not capable of sustaining growth in the absence of threonine, and therefore a working cell bank was prepared. Panel b describes the shake flask experiments and progression followed to evaluate the strain’s ability to be evolved from threonine supplementation to glycine supplementation. When 20G culture was inoculated from threonine supplemented medium to glycine only supplemented medium, no growth was observed up to 14d post-inoculation (2 samples per day measuring OD_600_). On day 14, a shake flask culture of 20G only supplemented with glycine, was then supplemented with 100 mg L**^−^** ^1^ threonine, and growth was immediately restored. It was therefore concluded that the mutant 20G was incapable of catalyzing glycine to threonine to satisfy threonine cellular demands, given that threonine synthesis was interrupted with the deletion of *thr1*. This experimental conclusion further supports that *Gly1* encoding threonine aldolase, originally believed to catalyze the conversion of glycine to threonine, catalyzes the reverse reaction and thus cannot meet threonine cellular demands from glycine pools.
(PNG)
