# Supplementary figures and images for: Industrial Systems Biology of Saccharomyces cerevisiae Enables Novel Succinic Acid Cell Factory
Source: PLoS One. 2013 Jan 21;8(1):e54144. doi: 10.1371/journal.pone.0054144 (PMC3549990; doi:10.1371/journal.pone.0054144)

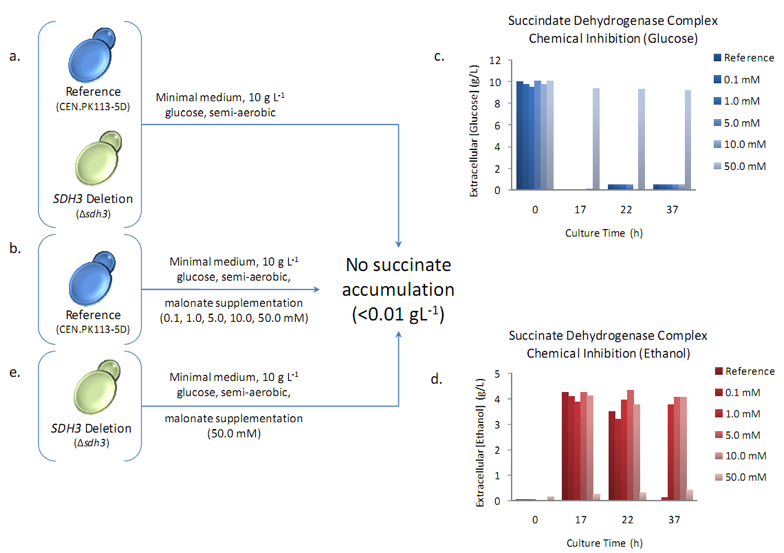

Supplement: Figure S1 — Inhibition of the succinate dehydrogenase complex with malonate supplementation in shake flask cultures was evaluated. The reference and Δsdh3 strain, previously described [17], were cultured in minimal media supplemented with 10 g L−1 glucose and no succinate accumulation was detected (Panel a). The reference strain was cultured with 0.1, 1.0, 5.0, 10.0, and 50.0 mM malonate supplementation. Under no supplementation conditions succinate accumulation was observed (Panel b). In order to confirm that the concentration of malonate in the culture was effectively inhibiting succinate dehydrogenase activity, residual ethanol in the culture broth was monitored. Succinate dehydrogenase activity, as previously described, catalyzes the conversion of succinate to fumarate with net production of protonated ubquinone. Ethanol is a carbon source readily catabolized by S. cerevisiae using respiro-fermentative pathways and requiring succinate dehydrogenase activity. Panel c shows the residual glucose concentration in the culture broth at 0, 17, 22, and 37h post-inoculation for no supplementation of malonate (reference) and then 0.1, 1.0, 5.0, 10.0, and 50.0 mM malonate supplementation. These growth profiles were generated using the reference strain (CEN.PK113-7D). As expected, full catabolism of glucose was observed at all malonate concentrations with the exception of 50.0 mM, thereby considered an upper limit. Similarly, in panel d, is the ethanol concentration in the culture broth for the same malonate concentrations and sample times. At 37 h, as expected, the reference strain had consumed nearly all ethanol produced during the glucose consumption phase. Malonate concentrations of 1.0, 5.0, and 10.0 mM malonate resulted in significant ethanol respiration inhibition compared to no supplementation and 0.1 mM malonate, confirming that respiro-fermentative catabolism was inhibited. Under no circumstances was succinate accumulation observed. Furthermore, the Δsdh3 strain was supplemen [file pone.0054144.s001.png]

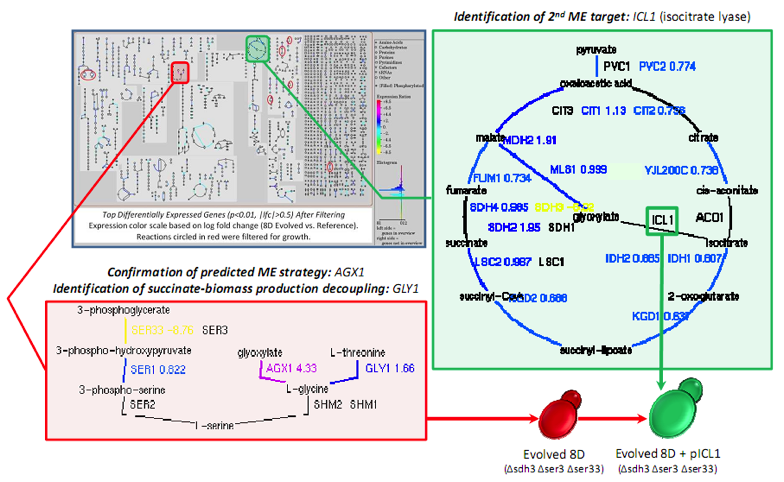

Supplement: Figure S2 — A total of 1964 genes were submitted to the Saccharomyces Genome Database tool, Pathway Expression Viewer. The resulting Pathway Expression map shows the relative log-fold change of all S. cerevisiae metabolic reactions (Evolved 8D vs. Reference). Three key results are high-lighted from the transcriptome. First, isocitrate lyase (ICL1) was amongst the few genes not up-regulated in the Evolved 8D strain, thereby becoming a 2nd round metabolic engineering target. Second, alanine:glyoxylate aminotransferase (AGX1) was 4.3 log-fold higher in the Evolved 8D strain, confirming the predicted model-guided strategy of up-regulated glycine formation from glyoxylate pools. Third, threonine aldolase (GLY1) was 1.6 log-fold higher in the Evolved 8D strain. The genome-scale model reconstruction used for predictions annotated Gly1p as catalyzing the reversible conversion of threonine to glycine. This reaction has since been shown to be irreversible, converting threonine to glycine, consuming equimolar acetaldehyde. The transcriptome data suggests that the Evolved 8D strain demonstrated de-coupling of succinate and biomass production because alternative reactions (e.g., Gly1p) were supplying glycine pools. (PNG) [file pone.0054144.s002.png]

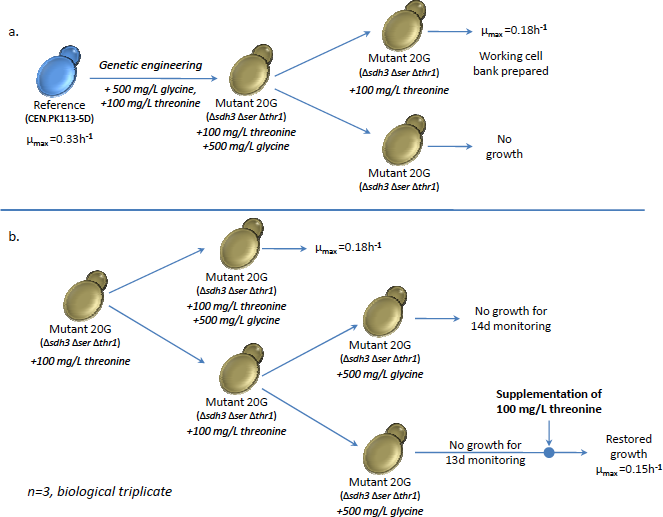

Supplement: Figure S3 — Panel a briefly describes the mutant construction of 20G, Δsdh3 Δser3 Δthr1 , from the reference strain and initially supplemented with 100 mg L−1 threonine and 500 mg L−1 glycine to satisfy the resulting auxotrophies. All growth challenges were evaluated in shake flasks supplemented with minimal medium, 300 mg L− 1 uracil, 10 g L− 1 glucose, and either threonine and/or glycine added, as indicated. The mutant 20G was not capable of sustaining growth in the absence of threonine, and therefore a working cell bank was prepared. Panel b describes the shake flask experiments and progression followed to evaluate the strain’s ability to be evolved from threonine supplementation to glycine supplementation. When 20G culture was inoculated from threonine supplemented medium to glycine only supplemented medium, no growth was observed up to 14d post-inoculation (2 samples per day measuring OD600). On day 14, a shake flask culture of 20G only supplemented with glycine, was then supplemented with 100 mg L− 1 threonine, and growth was immediately restored. It was therefore concluded that the mutant 20G was incapable of catalyzing glycine to threonine to satisfy threonine cellular demands, given that threonine synthesis was interrupted with the deletion of thr1. This experimental conclusion further supports that Gly1 encoding threonine aldolase, originally believed to catalyze the conversion of glycine to threonine, catalyzes the reverse reaction and thus cannot meet threonine cellular demands from glycine pools. (PNG) [file pone.0054144.s003.png]
